# Elucidation of the mechanisms of fluconazole resistance and repurposing treatment options against urinary *Candida* spp. isolated from hospitalized patients in Alexandria, Egypt

**DOI:** 10.1186/s12866-024-03512-0

**Published:** 2024-10-01

**Authors:** Hend Zeitoun, Rawan A. Salem, Nadia M. El-Guink, Nesrin S. Tolba, Nelly M. Mohamed

**Affiliations:** 1 Department of Microbiology and Immunology, Faculty of Pharmacy, Alexandria University, El-Khartoom Square, Azarita, Alexandria Egypt; 2 Department of Pathology, Medical Research Institute, Alexandria University, Alexandria, Egypt

**Keywords:** *Candida* spp., Urinary tract infections, Fluconazole resistance, *In vivo*, Real-time PCR, Repurposing, Colistin, Checkerboard titration technique

## Abstract

**Background:**

The incidence of fungal urinary tract infections (UTIs) has dramatically increased in the past decades, with *Candida* arising as the predominant etiological agent. Managing these infections poses a serious challenge to clinicians, especially with the emergence of fluconazole-resistant (FLC-R) *Candida* species. In this study, we aimed to determine the mechanisms of fluconazole resistance in urinary *Candida* spp. isolated from hospitalized patients in Alexandria, Egypt, assess the correlation between fluconazole resistance and virulence, and explore potential treatment options for UTIs caused by FLC-R *Candida* strains.

**Results:**

Fluconazole susceptibility testing of 34 urinary *Candida* isolates indicated that 76.5% were FLC-R, with a higher prevalence of resistance recorded in non-*albicans Candida* spp. (88.9%) than in *Candida albicans* (62.5%). The calculated Spearman’s correlation coefficients implied significant positive correlations between fluconazole minimum inhibitory concentrations and both biofilm formation and phospholipase production. Real-time PCR results revealed that most FLC-R isolates (60%) significantly overexpressed at least one efflux pump gene, while 42.3% significantly upregulated the *ERG11* gene. The most prevalent mutation detected upon *ERG11* sequencing was G464S, which is conclusively linked to fluconazole resistance. The five repurposed agents: amikacin, colistin, dexamethasone, ketorolac, and sulfamethoxazole demonstrated variable fluconazole-sensitizing activities in vitro, with amikacin, dexamethasone, and colistin being the most effective. However, the fluconazole/colistin combination produced a notable reduction (49.1%) in bladder bioburden, a 50% decrease in the inflammatory response, and tripled the median survival span relative to the untreated murine models.

**Conclusions:**

The fluconazole/colistin combination offers a promising treatment option for UTIs caused by FLC-R *Candida*, providing an alternative to the high-cost, tedious process of novel antifungal drug discovery in the battle against antifungal resistance.

**Supplementary Information:**

The online version contains supplementary material available at 10.1186/s12866-024-03512-0.

## Background

Despite being one of the major opportunistic fungi causing high morbidity and mortality worldwide, the *Candida* genus has long been underestimated as a public health threat [1, 2]. Members of this genus are currently responsible for an array of infections, including oral and cutaneous candidiasis, vaginitis, candidemia, systemic and urinary tract infections (UTIs) [2]. The incidence of fungal UTIs has dramatically increased in recent decades, with *Candida albicans* arising as the predominant etiological agent of these UTIs [3, 4]. Nevertheless, in the past years, a progressive shift to non-*albicans Candida* (NAC) UTIs caused by *C. glabrata*,* C. tropicalis*, and *C. krusei* has been noticed [4]. The UTIs caused by *Candida* spp. can be predisposed by old age, female sex, prolonged hospitalization, admission to intensive care units (ICUs), or the use of immunosuppressants, radiotherapy, broad-spectrum antibiotics, and urinary tract instruments [5, 6]. *Candida* utilizes multiple virulence factors, including the secretion of extracellular hydrolytic enzymes and biofilm formation, to colonize and invade the urinary tract [7]. The hydrolytic enzymes promote adherence to host tissue, host cell lysis, invasion of mucosa and blood vessels, and circumvention of the host’s immune response [7]. Among the most important enzymes are proteinases, encoded by *Sap1*–*Sap10* genes, which degrade both structural and immunologic proteins [7, 8]. Additionally, phospholipases, regulated mainly by *PLB1* and *PLB5* genes, hydrolyze glycerophospholipids compromising the integrity of the host cell membranes [7, 8]. On the other hand, biofilm formation, promoted by *Hwp1* and *Als3* genes, is not only crucial for the growth of *Candida* on medical devices such as urinary catheters but also reduces the susceptibility of yeast cells to antifungals [7, 8].

The diagnosis of *Candida* UTIs presents a serious challenge to clinicians, due to the lack of a definitive method for the immediate differentiation of contamination, colonization, and actual infection. Therefore, a systematic approach is prudent [9]. Since contamination of urine samples is common, it is advisable to repeat the urine culture and ensure the collection of a clean-catch midstream sample, to rule out contamination [10]. Colonization, on the other hand, refers to the asymptomatic adherence of *Candida* to catheters or other foreign bodies in the urinary tract, often resulting in high concentrations of *Candida* in urine cultures [11]. In patients with indwelling catheters, it is recommended to replace the catheter before obtaining a second urine sample to rule out the possibility of colonization [10].

Infectious Diseases Society of America (IDSA) guidelines recommend the administration of fluconazole (FLC) as first-line therapy for symptomatic *Candida* UTIs due to its favorable oral bioavailability and its capacity to achieve high concentrations in urine [12]. This triazole antifungal inhibits lanosterol 14α-demethylase, encoded by the *ERG11* gene, hence it hinders the ergosterol biosynthesis pathway and disrupts the cell membrane, impeding the proliferation of *Candida* [13]. Alarmingly, recent observational studies detected the growing emergence of FLC resistance in *C. tropicalis* and *C. albicans* isolated from urine, reaching rates equivalent to 19% and 8%, respectively [14, 15]. The situation in Egypt appears to be even more problematic, where El Said et al. stated that 55.7% of their urinary *Candida* isolates were FLC-resistant (FLC-R), most of which belonged to *C. glabrata*, followed by *C. krusei*, *C. tropicalis*, and *C. albicans* [16].

The development of FLC resistance among *Candida* spp. may arise due to the overexpression of the *ERG11* gene leading to an increased ergosterol production or due to mutations in the *ERG11* gene, inducing amino acid substitutions and minimizing the FLC binding efficiency to its target enzyme [17]. Apart from mechanisms involving the ergosterol biosynthetic pathway, the efflux of the antifungal agent is considered the most common cause of FLC resistance in *Candida* [18]. Many FLC-R *Candida* isolates overexpress efflux pumps, including ATP-Binding Cassette (ABC) transporters encoded by Candida drug resistance-1 (*CDR1*) and Candida drug resistance-2 (*CDR2*) genes, or Major Facilitator Superfamily (MFS) transporters encoded by multidrug resistance-1 (*MDR1*) gene [17, 19]. This leads to a failure in the intracellular accumulation of FLC, resulting in resistance [19]. An infrequent mechanism of resistance is the loss-of-function mutation in *ERG3*, which inactivates Δ5,6-sterol desaturase and allows the fungal cell to bypass the production of toxic methylated sterols, minimizing the effect of FLC [19].

The elevated incidence of *Candida* infections, in addition to the increase in the rate of FLC resistance, warrant the development of novel antifungal drugs; however, limited financial resources are allocated to this field of research. Hence, repurposing drugs of disparate clinical indications, yet demonstrating weak antifungal activity, is a promising solution to this dilemma [2]. Among the drugs reported to possess anti-*Candida* activity are the anti-inflammatory dexamethasone (DEX) and ketorolac tromethamine (KT), the anti-hyperlipidemic atorvastatin, as well as the antibacterial amikacin (AK), colistin sulfate (COL), and sulfamethoxazole (SMX) [2, 20–24]. Despite the availability of ample global data analyzing the molecular mechanisms of FLC resistance, there is a scarcity of literature originating from the African region and developing countries. In this study, we aimed to determine the prevalence of FLC resistance among urinary *Candida* spp. isolated from hospitalized patients in Alexandria, Egypt, while elucidating the underlying mechanisms of this resistance. In addition, the repurposing strategy is explored, where the efficacy of AK, COL, DEX, KT, and SMX in increasing the susceptibility of FLC-R *Candida* isolates towards FLC is tested through selected in vitro and in vivo techniques.

## Results

### Identification of *Candida* isolates and phenotypic detection of their virulence attributes

#### Isolates identity to the species level and their biofilm-forming ability

**Fig. 1:**
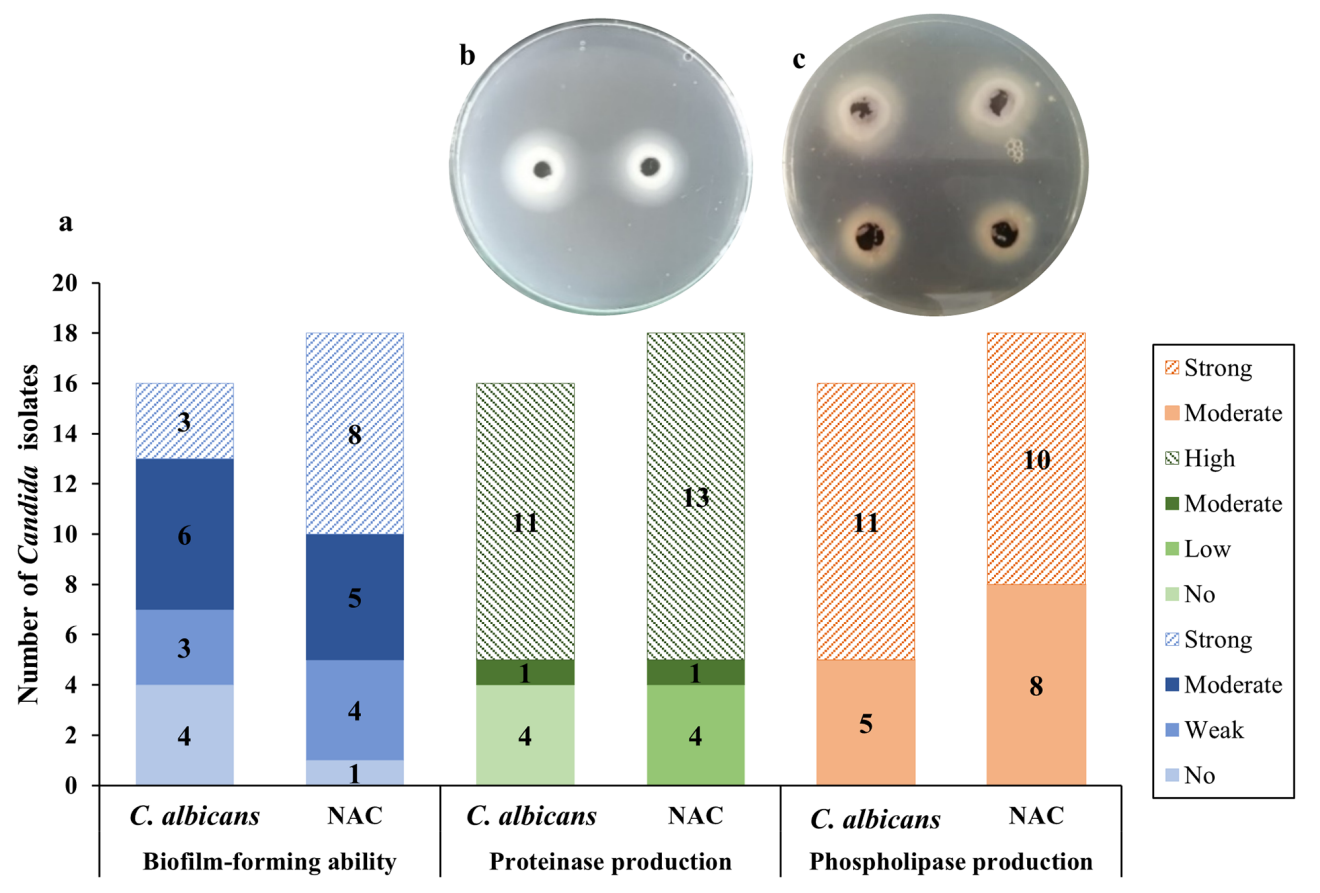
**a** Prevalence of biofilm formation, proteinase, and phospholipase production among *C. albicans* and NAC isolates; **b** Isolate CT2 showing high proteinase production (Pz value = 0.35); **c** Isolates CD1 (on top, Pz value = 0.53) and CA16 (at the bottom, Pz value = 0.4) demonstrating strong phospholipase activity. *NAC* refers to non-*albicans Candida* spp. isolates

The phenotypic tests used for identification, including germ tube formation, and Tween 80 opacity tests, as well as the confirmatory Vitek^®^, classified the 34 collected isolates into 16 *C. albicans* (designated as CA1 to CA16), 10 *C. tropicalis* (CT1 to CT10), six *C. glabrata* (CG1 to CG6), a *C. famata* (CF1), and a *C. dubliniensis* (CD1) (Additional file 1: Table S1). Representative positive results of the phenotypic tests used for identification are shown in Additional file 2: Fig. S1. The assessment of the biofilm-forming ability in these isolates using the microtiter plate (MTP) method revealed more prominent biofilm formation in NAC than in *C. albicans* isolates. This is evidenced by strong biofilm formation in eight (44.4%) NAC isolates compared to three (18.8%) *C. albicans* isolates. In addition, a single NAC isolate (5.6%) was shown to be a non-biofilm former, as opposed to four (25%) *C. albicans* isolates (Fig. 1).

#### Extracellular enzymes production

The ability of the tested isolates to produce two extracellular hydrolytic enzymes, proteinase, and phospholipase, was evaluated. Higher proteinase activity was detected among NAC isolates, with 13 (72.2%), one (5.6%), and four (22.2%) isolates exhibiting high, moderate, and low proteinase production, respectively. On the other hand, 11 (68.8%) *C. albicans* isolates were high proteinase producers, a single isolate (6.3%) was a moderate producer, and 25% of the isolates were non-producers. All *Candida* isolates demonstrated positive phospholipase activity, with no major differences detected between *C. albicans* and NAC isolates. *C. albicans* isolates were segregated into 11 (68.8%) strong phospholipase producers and five (31.3%) moderate producers, while NAC isolates were classified into 10 (55.6%) strong producers and eight (44.4%) moderate producers (Fig. 1).

### Fluconazole susceptibility testing

The sensitivity of the isolates to FLC was determined using the disk diffusion (DD) and broth microdilution (BMD) techniques. The results demonstrated excellent alignment, except for three isolates, where the BMD method yielded a lower degree of FLC susceptibility than the DD method, classifying the two *C. albicans* isolates, CA13, and CA14, as susceptible-dose-dependent (SDD) rather than susceptible (S), and the CG3 isolate as resistant (R) rather than SDD (Additional file 1: Table S2). The minimum inhibitory concentration (MIC) of FLC against the collected isolates ranged between 1 and > 1000 µg/mL. A percentage of 76.5% of *Candida* isolates were FLC-R, with a higher prevalence of FLC resistance in NAC isolates (88.9%) than in *C. albicans* isolates (62.5%) (Table 1). Notably, all the *C. tropicalis* isolates collected in this study were FLC-R.

**Table 1:** Fluconazole susceptibility in different *Candida* spp. isolates

Candida spp. (*n*)	FLC-*R* isolates *n* (%)	MIC_50_^a^	MIC_90_^b^	MIC range (µg/mL)
Total (34)	26 (76.5)	128	1000	1 - >1000
*C. albicans* (16)	10 (62.5)	32	> 1000	1 - >1000
NAC (18)	16 (88.9)	128	512	1 - >1000
*C. tropicalis* (10)	10 (100)	128	256	128 - >1000
*C. glabrata* (6)	5 (83.3)	64	512	32–512
*C. famata* (1)	1 (100)	NA	NA	NA
*C. dubliniensis* (1)	0 (0)	NA	NA	NA

^a^ The minimum concentration at which 50% of the tested isolates were inhibited. ^b^ The minimum concentration at which 90% of the tested isolates were inhibited. *FLC-R* fluconazole-resistant, *NAC*** non-*albicans Candida* spp**., *NA* not applicable

### Antifungal activity of repurposing agents

The growth-inhibitory activity of the repurposing agents was tested against the 26 FLC-R *Candida* isolates. The MICs of AK, KT, SMX, and DEX against these isolates were ≥ 16,384, >2048, ≥ 2048, and ≥ 2000 µg/mL, respectively (Additional file 1: Table S3). However, COL demonstrated variable MIC values against the tested isolates, with MIC_50_ and MIC_90_ values equivalent to 1024 and > 2048 µg/mL, respectively.

### Correlation between the assessed virulence factors and FLC susceptibility profile

**Fig. 2:**
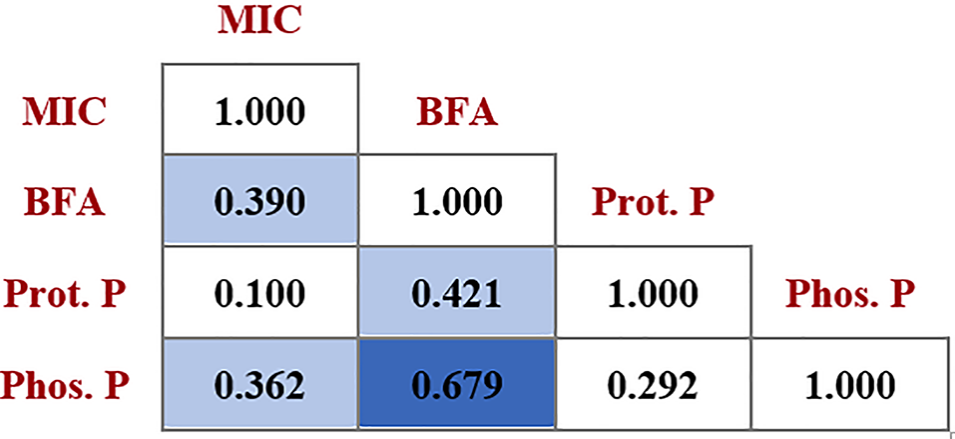
Correlation matrix showing Spearman’s correlation coefficients (r_s_) for the investigated virulence factors and the MIC of FLC calculated for 34 tested *Candida* spp. isolates. Light-blue-colored cells indicate significance at a *p*-value < 0.05, while dark-blue-colored cells indicate significance at a *p*-value < 0.01. “BFA” indicates the biofilm-forming ability, “Prot. P” refers to proteinase production, while “Phos. P” represents phospholipase production

The correlations between the three assessed virulence attributes and those between the MICs of FLC against the isolates and their virulence were investigated through the calculation of Spearman’s correlation coefficient (r_s_) values. Results indicated a significant positive correlation between FLC resistance and both biofilm formation and phospholipase production and a similar correlation between biofilm formation and proteinase production. In addition, a highly significant positive correlation was detected between biofilm formation and phospholipase production (Fig. 2).

### Molecular characterization of FLC resistance mechanisms

#### Quantification of *ERG11* gene and efflux pump genes: *MDR1*, *CDR1*, and *CDR2*

**Fig. 3:**
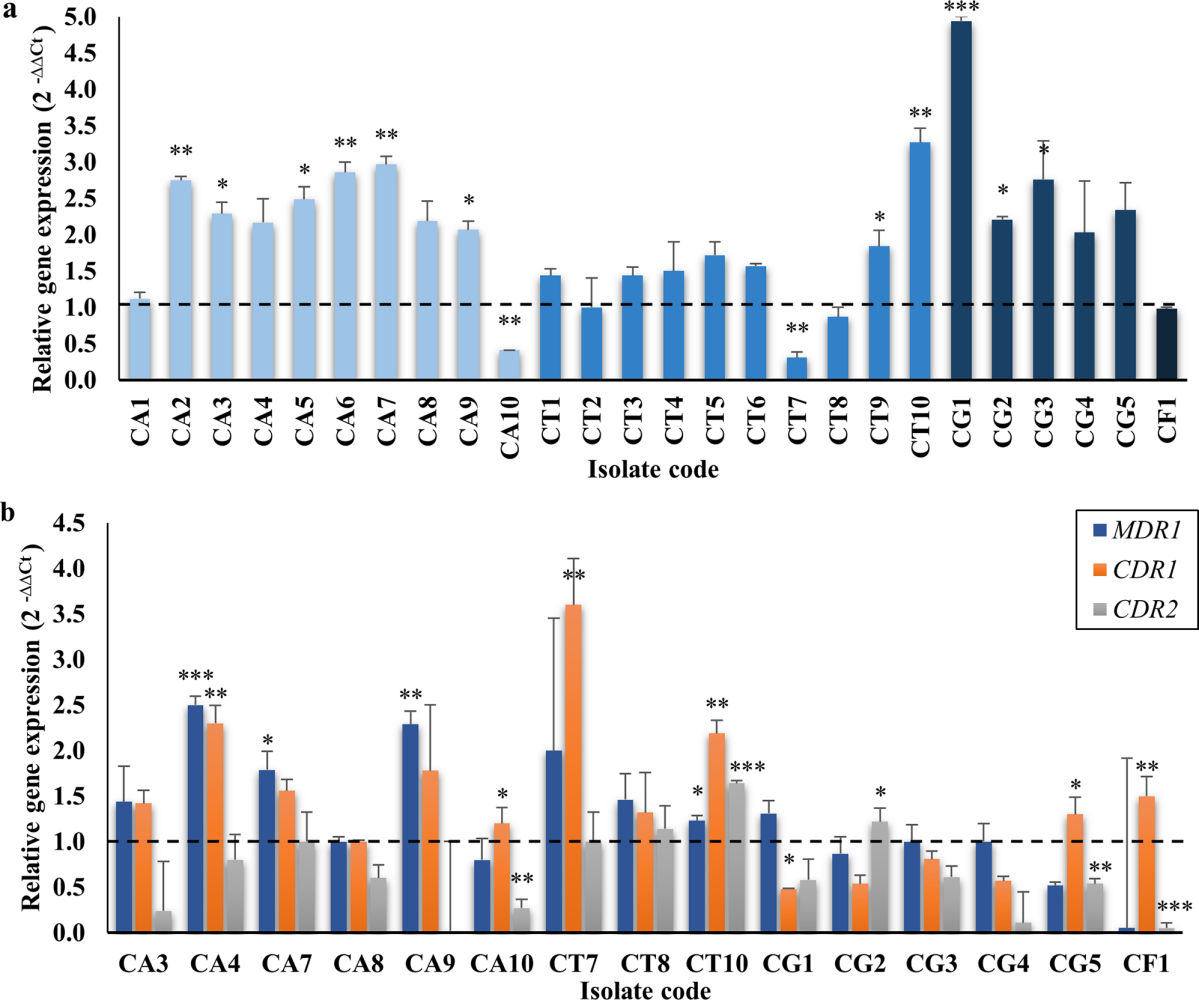
Expression levels of **a ***ERG11* gene and **b ***MDR1*,*CDR1*, and *CDR2* genes in FLC-R isolates relative to *C. albicans* ATCC 10231. The error bars represent the standard errors of the mean. The *p*-values indicate the significance of fold change at * *p* < 0.05, ** *p* < 0.01, and *** *p* < 0.001. CA indicates *C. albicans* isolates, CT stands for *C. tropicalis* isolates, CG refers to *C. glabrata* isolates, and CF stands for *C. famata* isolate

Real time-PCR (RT-PCR) was performed to determine the expression levels of the *ERG11* gene in the FLC-R isolates and those of efflux pump genes in 15 randomly selected FLC-R isolates. Analysis of the results indicated that 21 (80.8%) isolates overexpressed the *ERG11* gene relative to the FLC-sensitive (FLC-S) *C. albicans* ATCC 10231. This upregulation ranged between 1.1- and 4.9-fold and was statistically significant in 11 (52.4%) of these isolates. On the other hand, eight (53.3%) isolates demonstrated upregulation of the *MDR1* gene ranging between 1.2- and 2.5-fold, which was significant in four (50%) of these isolates. The genes encoding the ABC transporters, *CDR1*, and *CDR2*, were overexpressed in 10 (66.7%) and three (20%) isolates, respectively. The overexpression of *CDR1* was statistically significant in six (60%) isolates, and that of *CDR2* in two (66.7%) isolates **(**Fig. 3**)**. Four isolates, CA4, CA7, CA9, and CG2, showed a statistically significant upregulation of two genes, whereas a single isolate, CT10, significantly overexpressed the four investigated genes. Of note, all the above-mentioned isolates displayed high phenotypic resistance to FLC with MIC values ranging from 32 to 1000 µg/mL.

#### PCR amplification and sequencing of the *ERG11* gene

Before sequencing, the sizes of the PCR products for the *ERG11* gene were confirmed through agarose gel electrophoresis, and the results are illustrated in Additional file 2: Fig. S2. Sequencing analysis of the *ERG11* gene in selected *C. tropicalis* isolates revealed that the most frequently detected amino acid substitution was G464S (Table 2). This substitution was identified in all the tested *C. tropicalis* isolates and was located in hotspot III (amino acids 405–488). Other detected substitutions were newly observed, did not belong to any of the reported hotspots, and each of them appeared only once.

**Table 2:** *ERG11* sequence analysis in investigated *C. tropicalis* isolates

Candida spp. isolate	CT1	CT2	CT3	CT8
Amino acid substitution(s)	F28H, F30Y,	**G464S**	**G464S**	**G464S**
	**G464S**			

Amino acid substitutions in bold were previously reported to be associated with fluconazole resistance. *CT**** C. tropicalis***, amino acid abbreviations: *S* serine, *F* phenylalanine, *H* histidine, *Y* tyrosine, *G* glycine

### In vitro assessment of FLC interaction with potential repurposing agents

#### Checkerboard titration technique

To evaluate the efficiency of the investigated repurposing agents in combination with FLC, the checkerboard titration technique was applied to four FLC-R isolates representing the different species encountered in the current study. The results demonstrated that AK and COL were the most promising agents, as both showed synergy when combined with FLC against all the tested isolates **(**Table 3**)**. Synergy was detected, as well, when combining DEX with FLC in 75% of the tested isolates. The least successful combinations were those of KT or SMX with FLC, where synergy was displayed in 50% of the tested isolates.

**Table 3:** FIC index for the combined activity of fluconazole with investigated repurposing agents against representative *Candida* spp. isolates

*Candida* spp. isolate	ƩFICI (Interpretation)				
	AK	COL	DEX	KT	SMX
CA1	0.25 (Syn)	0.28 (Syn)	1.00 (Add)	1.00 (Add)	2.00 (Ind)
CT1	0.25 (Syn)	0.28 (Syn)	0.50 (Syn)	1.00 (Add)	2.00 (Ind)
CG1	0.32 (Syn)	0.50 (Syn)	0.25 (Syn)	0.26 (Syn)	0.25 (Syn)
CF1	0.09 (Syn)	0.06 (Syn)	0.16 (Syn)	0.10 (Syn)	0.07 (Syn)

*ΣFICI* fractional inhibitory concentration index, *AK* amikacin, *COL* colistin sulfate, *DEX* dexamethasone, *KT* ketorolac tromethamine, *SMX* sulfamethoxazole, *CA ****C. albicans***,* CT ****C. tropicalis***,* CG ****C. glabrata***,* CF ****C. famata***, *Syn* synergism, *Add* additivity, *Ind* indifference

#### Resistance modulation assay

**Fig. 4:**
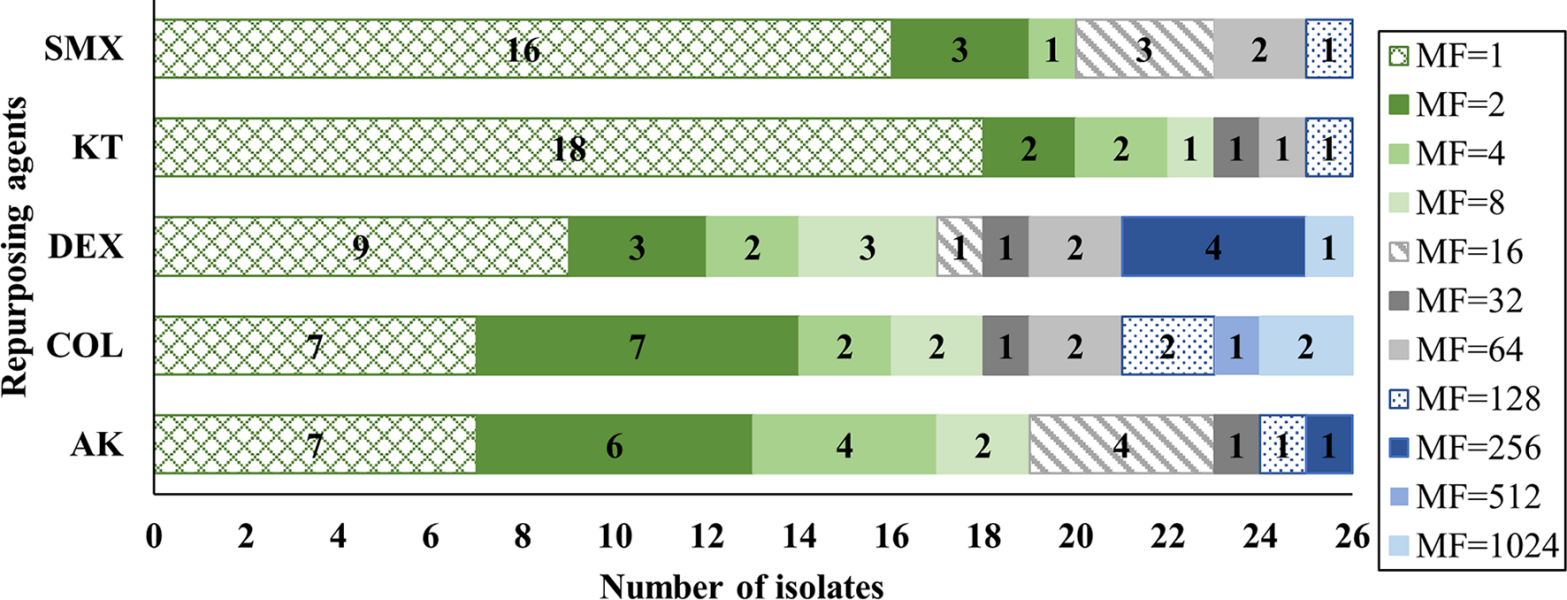
Fluconazole-potentiating efficacy of AK, COL, DEX, SMX, and KT (used at concentrations equivalent to 0.25X MIC) against the FLC-R *Candida* isolates. MF represents the calculated modulation factor for each agent

The modulation factor (MF) of AK, COL, DEX, SMX, and KT against the 26 FLC-R isolates was determined (Additional file 1: Table S4), and agents with MF > 2 were considered to have an FLC-potentiating activity. A pronounced modulation of the antifungal activity of FLC was exerted by the repurposing agent, DEX, which was able to sensitize 54% of the tested isolates with an MF ranging from 4 to 1024 (Fig. 4). Significant reductions in the MIC values of FLC were obtained as well by the modulatory activities of AK and COL, both promoting FLC activity in 50% and 46% of the isolates with an MF ranging from 4 to 250 and from 4 to 1000, respectively. Meanwhile, SMX and KT were found to be the least effective agents, failing to sensitize 73% and 77% of the isolates to FLC, respectively.

#### Rhodamine efflux assay

**Fig. 5:**
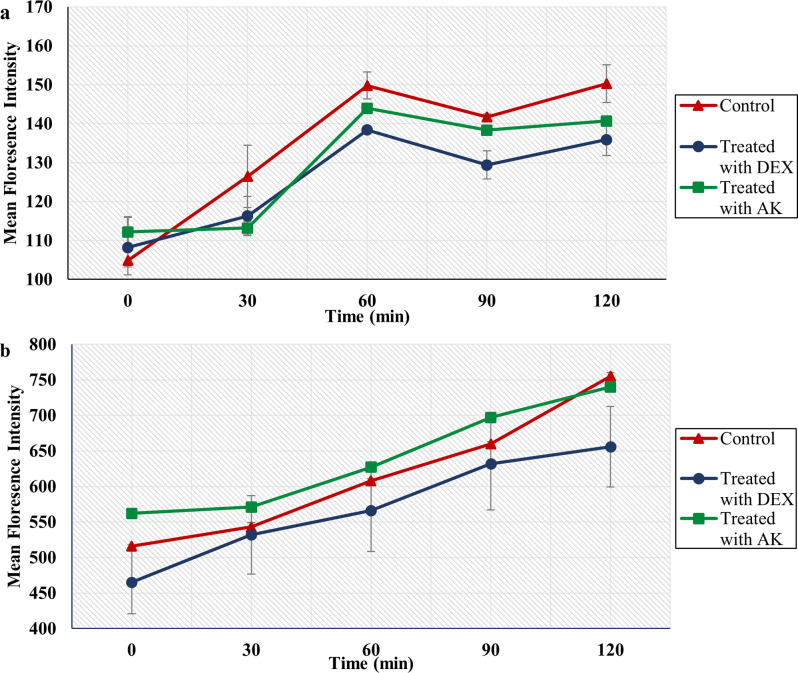
Inhibitory effects of AK (4096 µg/mL) and DEX (250 µg/mL) on rhodamine efflux in **a ***C. albicans* isolate, CA10, and **b ***C. tropicalis* isolate, CT7. The error bars represent the standard errors of the mean

The rhodamine efflux assay was used to inspect the inhibitory effects of AK and DEX on the activity of efflux pumps, using two FLC-R isolates, a *C. albicans*, and an NAC isolate. Two hours post-treatment of isolate CA10, AK, and DEX produced a 6.4% and a high statistically significant reduction of 9.6% in the mean fluorescence intensity relative to the control group (*p*-value = 0.002), respectively. A noteworthy observation was that AK demonstrated the highest percentage reduction (10.5%) after 30 min. On the other hand, AK and DEX produced a 2% and 13% reduction in the mean fluorescence intensity after 120 min when applied to isolate CT7, respectively (Fig. 5). Hence, the inhibitory effect of DEX on efflux pump activity was 1.5X that of AK on isolate CA10 and 6.5X on isolate CT7.

### In vivo assessment of combined therapy

#### Organ bioburden experiment

**Fig. 6:**
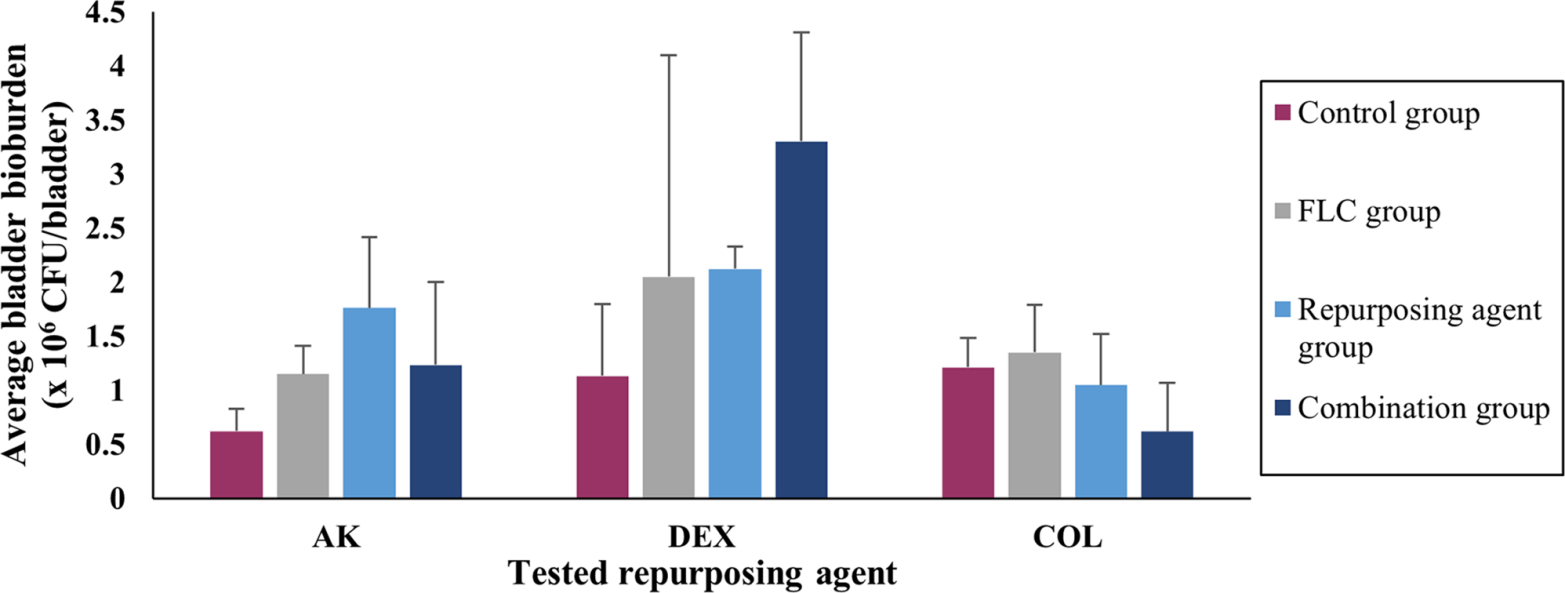
Average bioburden of *C. albicans*, CA9 isolate, in the dissected bladders of challenged mice in the control (200 µL water for injection), FLC (50 mg/kg), repurposing agent (40 mg/kg AK, 0.6 mg/kg DEX, or 5 mg/kg COL), and combination groups. Error bars represent standard errors of the mean

The in vivo efficacies of AK, DEX, and COL were assessed against isolate CA9 using the bladder bioburden experiment. This isolate was selected as it demonstrated high FLC resistance (MIC = 1000 µg/mL) and favorable MFs. Out of the tested combinations, the FLC/COL combination produced the most prominent reduction in the bladder fungal burden (49.1%) relative to the control group. This reduction was clearly due to the combined effect of FLC/COL, since individually, FLC did not reduce the count relative to the control group, while COL displayed a weak bioburden reduction equivalent to 13.7% upon its administration to the challenged mice (Fig. 6).

#### Animal survival experiment

**Fig. 7:**
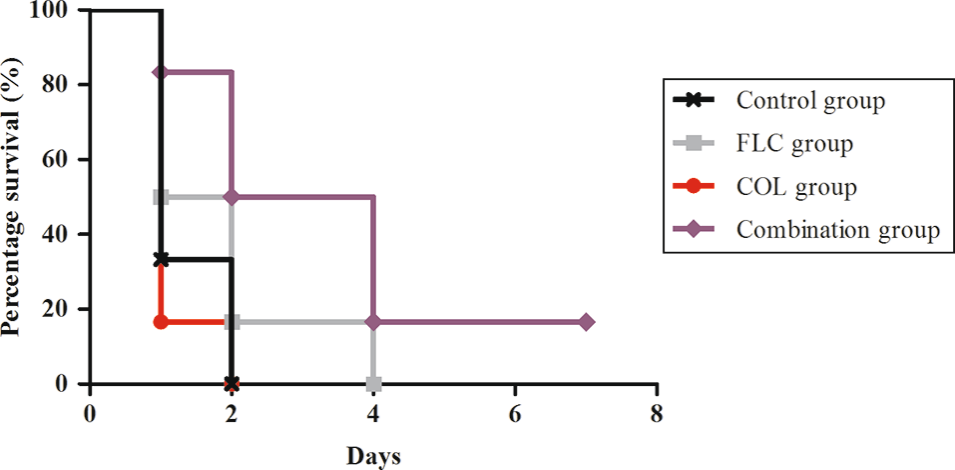
The survival rate of mice in the control (200 µL water for injection), FLC (50 mg/kg), COL (5 mg/kg), and FLC/COL combination groups over one week

Intraperitoneally (IP) infected mice with a lethal dose of isolate CA9 were monitored for a week to examine the effect of the leading combination (FLC/COL) on their survival rate. By the end of day two of observation, the mice in the control and COL groups died, while the FLC and the combination groups displayed a percentage survival of 16.7% and 50%, respectively. Additionally, by the end of the week, all groups had no survivors, except for the combination group which demonstrated a survival rate of 16.7% (Fig. 7). Overall, there was a significant difference in the survival curves of the groups (*p*-value = 0.0364), and the median survival in the combination group (3 days) was double that of the FLC group (1.5 days) and triple that of the control and COL groups (1 day).

#### Histopathology

**Fig. 8:**
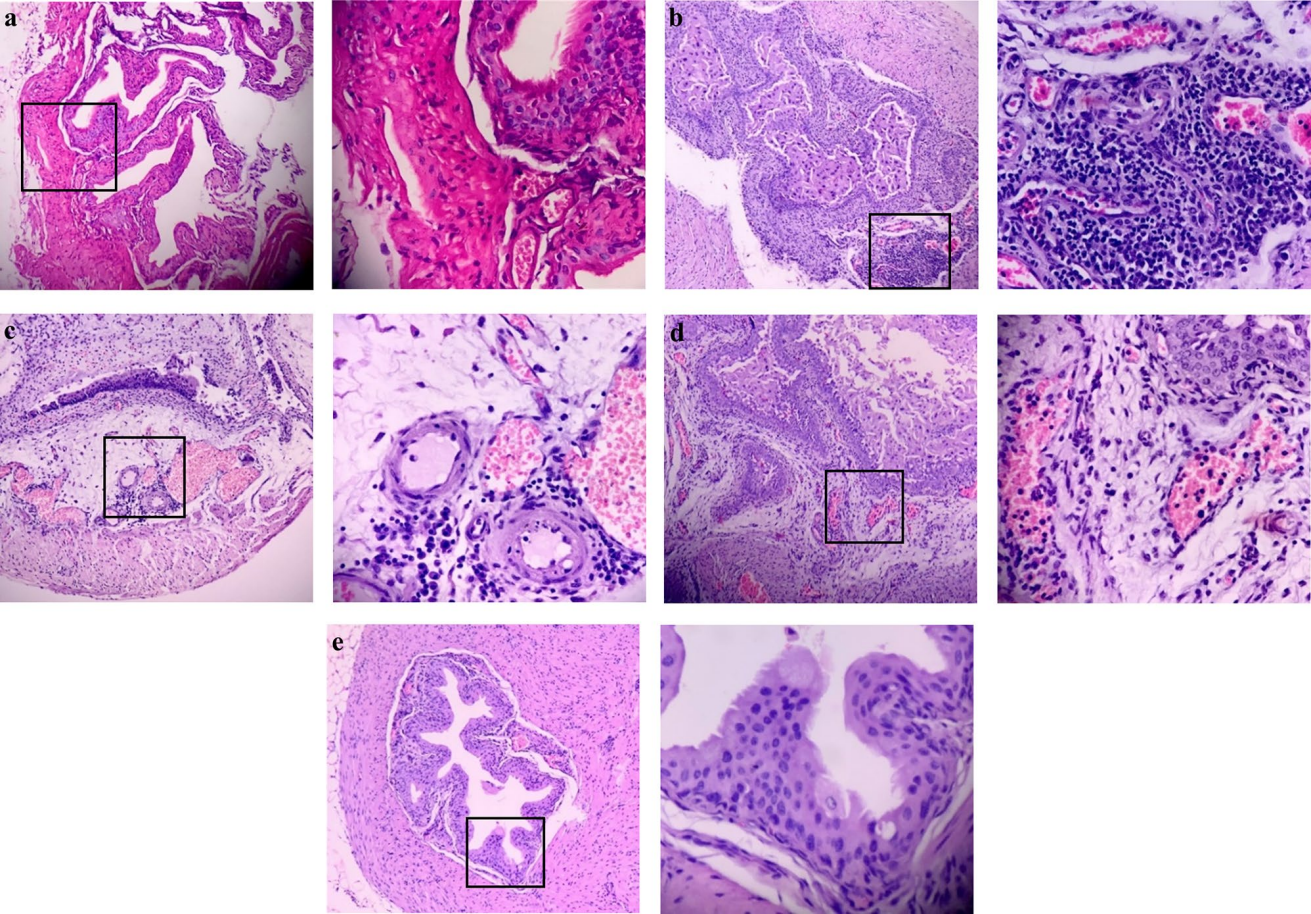
Histology of the urinary bladders isolated from **a** an uninfected mouse showing intact mucosa formed of 4–6 layers of transitional cells resting upon lamina propria and muscularis propria devoid of inflammatory cells (100X). Inset depicting the absence of inflammatory components (400X); **b** Infected untreated mouse demonstrating shedding of epithelial cells, focal expansion of lamina propria by inflammatory components, and congested blood vessels (100X). Inset showing the inflammatory components; mainly lymphocytes, plasma cells, and neutrophils, as well as severely congested blood vessels (400X); **c** Fluconazole-treated mouse (50 mg/kg) showing shedding of epithelial cells with complete loss of epithelium in some areas, edema of lamina propria with evident inflammatory cell components and congested blood vessels (100X). Inset showing edematous lamina propria with perivascular infiltration by lymphocytes, plasma cells, and polymorphs as well as congested blood vessels (400X); **d** Colistin-treated mouse (5 mg/kg) showing shedding of epithelial cells, diffuse expansion of lamina propria by inflammatory components as well as congested blood vessels (100X). Inset depicting massive edema of lamina propria entangling inflammatory cells; mainly lymphocytes, plasma cells, and neutrophils as well as extensively congested blood vessels (400X); **e** Combination-treated mouse showing a near-normal appearance except for a few congested blood vessels (100X). Inset depicting intact epithelium with normal features of lamina propria and muscularis propria (400X)

To assess whether the FLC/COL combination effectively protected the bladder tissue in *Candida*-infected mice, an average score for the inflammatory responses was calculated for the control, monotherapy, and combination groups (Table 4). Treatment of infected mice with the FLC/COL combination resulted in a percentage reduction of 42.9% in the inflammatory response relative to the groups receiving FLC or COL monotherapy and a 50% reduction relative to the control group. The representative sections of the different groups shown in Fig. 8 ascertain the superior anti-inflammatory activity of the FLC/COL combination.

**Table 4:** Average inflammatory scores recorded in the bladders of *Candida*-infected mice in control (200 µL water for injection), FLC (50 mg/kg), COL (5 mg/kg), and FLC/COL combination groups

Group	Average inflammatory score ± SD
Untreated control group	2.00 ± 0.00
FLC group	1.75 ± 0.50
COL group	1.75 ± 0.50
Combination group	1.00 ± 0.82

*FLC* fluconazole, *COL* colistin sulfate, *SD* standard deviation

## Discussion

Candiduria is a frequent clinical finding characterized by the presence of *Candida* spp. in urine and is particularly encountered in hospital settings [9, 25]. In this study, *C. albicans* was the predominant culprit in candiduria cases (47.1%), followed by *C. tropicalis*, *C. glabrata*, *C. famata*, and *C. dubliniensis*. Collectively, NAC strains represented a higher recovery rate (52.9%) than *C. albicans*, aligning with multiple studies in Egypt and around the world, which report an epidemiological shift to NAC UTIs, especially those caused by *C. glabrata* and *C. tropicalis* [4, 16, 26–29].

The transition of *Candida* spp. from commensalism to pathogenesis is endorsed by a range of virulence factors, including evasion from the host’s immune response, adherence, biofilm formation, and secretion of extracellular hydrolytic enzymes [30]. Biofilms are implicated in an array of clinically relevant complications, especially in UTIs, where they develop on indwelling urinary catheters, promoting resistance to both antifungal agents and the host’s immune response [31]. In this study, a more prominent biofilm-forming ability was noted among NAC isolates, where 94.4% of these isolates were biofilm-formers, in contrast to 75% of the *C. albicans* isolates, which was corroborated by Ismail et al. in Egypt [27]. The hydrolytic enzymes secreted by *Candida* spp., proteinases, and phospholipases, participate in adherence, penetration, invasion, and destruction of tissues [31]. The tested NAC isolates demonstrated a higher proteinase positivity rate (100%) than *C. albicans* isolates (75%). Conversely, all isolates possessed phospholipase activity without apparent differences between the NAC and *C. albicans* isolates. Contradictory to our results, higher proteinase and phospholipase activities were reported in *C. albicans* than in NAC isolates [27, 32]. This discrepancy may be explained by the influence of comorbidities on hydrolytic enzyme activity. For instance, it has been reported that both diabetes and HIV infection significantly increase proteinase activity, while the latter also increases phospholipase activity [33–35].

The susceptibility of the isolates to FLC was assessed using DD and BMD techniques. Three isolates were categorized differently by the investigated methods, generating a good categorical agreement of 91.2% between both techniques. Aggarwal and Kashyap [36] reported a similar agreement of 86.8% and demonstrated higher FLC-resistance and susceptible-dose-dependence rates with BMD than the DD method, aligning with our results.

According to the Centers for Disease Control and Prevention (CDC), drug-resistant (formerly FLC-R) *Candida* was accountable for 34,800 cases and 1700 deaths in hospitalized patients in the USA during 2017, representing a serious threat to public health [37]. In the current study, 76.5% of the isolates were FLC-R, with a higher prevalence of resistance detected in NAC spp. Additionally, 83.3% of the *C. glabrata* isolates were resistant, and the remaining isolate was SDD. In accordance with our results, El Said et al. reported that 55.7% of their urinary *Candida* spp. collected from Giza, Egypt were FLC-R, with NAC isolates demonstrating higher FLC resistance than *C. albicans*. Moreover, 88.9% of *C. glabrata* isolates were FLC-R, and the rest were SDD [16]. In 2020, a study in Alexandria, Egypt, reported an FLC resistance rate of 100% among urinary *C. tropicalis*, a finding identical to ours [21]. The high FLC resistance rate documented in the current study may be attributed to FLC prescription for asymptomatic candiduria, patients’ non-compliance with a complete antifungal regimen, and the unprescribed use of FLC in the community.

The correlation between the three assessed virulence factors and that between FLC resistance and virulence attributes was investigated through the calculation of the r_s_ values. Significant positive correlations were detected between biofilm formation and the production of the two tested hydrolytic enzymes. In a study conducted in Egypt, a similar correlation was identified between biofilm formation and phospholipase production, inspiring the authors to suggest the use of anti-phospholipases to combat infections caused by biofilm-forming *Candida* isolates [38]. The simultaneous increase in biofilm formation and proteinase production was reported by Kadry et al. [39] and is speculated to originate from the role of proteolysis in *Candida* biofilm maintenance [40]. Moreover, we found that the FLC resistance showed a significant positive correlation with both biofilm formation and phospholipase production, a finding ascertained by Mohammadi et al. [41]. The positive correlation between biofilm formation and FLC resistance is postulated to arise from the FLC’s reduced ability to penetrate the biofilm matrix, exposing only the superficial layers to lethal FLC doses [31].

To elucidate the mechanisms of FLC resistance in the collected isolates, the expression levels of *ERG11* and efflux pump genes were determined in FLC-R isolates using RT-PCR. Drug efflux mediated through overexpression of efflux pumps was reported as the predominant cause of high levels of FLC resistance [18]. This applied to our results, where 60% of the tested FLC-R isolates significantly overexpressed at least one efflux pump gene, compared to 42.3% that demonstrated significant upregulation of the *ERG11* gene. Moreover, 33% of FLC-R *C. albicans* isolates overexpressed the *CDR1* gene, while none upregulated the *CDR2* gene. Similarly, Mane et al. [42] reported a higher percentage of FLC-R *C. albicans* isolates overexpressing *CDR1* than *CDR2*. On the other hand, overexpression of ABC transporters (encoded by *CDR1* and *CDR2*), but not MFS transporters (encoded by *MDR1*), was reported in *C. glabrata* isolates [43, 44]. This aligns with our results, where *CDR1*, and *CDR2* genes were overexpressed in 20% of the *C. glabrata* isolates, while none upregulated the *MDR1* gene. As for *C. tropicalis* isolates, 67%, 33%, and 33% significantly overexpressed *CDR1*, *CDR2*, and *MDR1* genes, respectively. The role of overexpression of *MDR1* and *CDR1* in the resistance of *C. tropicalis* to FLC has been consolidated by several studies [19, 45], however, that of *CDR2* was less frequently reported [46].

*ERG11* overexpression increases the lanosterol 14α-demethylase content in yeast cells, maintaining ergosterol synthesis and normal proliferation of *Candida* despite FLC treatment, thus resulting in reduced susceptibility to FLC [47]. Upregulation of the *ERG11* gene was detected in 60% of the FLC-R *C. albicans* and *C. glabrata* isolates, as well as 20% of the *C. tropicalis* isolates. The role of *ERG11* overexpression in the resistance developed by *C. albicans* and NAC isolates to FLC was confirmed by multiple studies [48–52]. Alternatively, *Candida* spp. can acquire FLC resistance through point mutations in the *ERG11* gene causing amino acid substitutions that reduce the FLC binding efficiency to the enzyme [17]. Most of the substitutions occur in three well-defined hotspot regions within the enzyme: amino acids 105–165, 266–287, and 405–488 [53]. Following the analysis of RT-PCR results, the mechanism of resistance in isolate CT2 was still unrevealed; hence, its *ERG11* gene was sequenced to detect mutations. Since *C. tropicalis* demonstrated the highest resistance rate in our study, three additional *C. tropicalis* isolates with statistically insignificant mechanisms of resistance were selected for *ERG11* sequencing. The most prevalent amino acid substitution detected was G464S (hotspot III), which is located below the heme group, hence it alters the heme environment decreasing its FLC affinity without affecting the enzyme activity [54]. G464S had been previously reported in an FLC-R urinary *C. tropicalis* isolate, conclusively linked to FLC resistance, and suggested as a predictive marker of azole resistance [45, 53, 55].

The innovative repurposing strategy was explored in an attempt to overcome FLC resistance among urinary *Candida* spp. The checkerboard titration technique was applied to four representative isolates for the preliminary assessment of the interaction of FLC with the repurposing agents, AK, COL, DEX, KT, and SMX. None of these agents possessed individual significant antifungal activity against FLC-R *Candida* isolates, as confirmed by their high MIC values. Despite that, AK and COL demonstrated synergy with FLC against 100% of the tested isolates. Dexamethasone was synergistic with FLC against 75% of the isolates, while KT and SMX showed synergism in only 50% of the isolates. Subsequently, a resistance modulation assay was used to investigate the FLC-potentiating activity of the repurposing agents against all the FLC-R isolates. Confirmatory to the checkerboard results, DEX, AK, and COL had an FLC-sensitizing effect on 54%, 50%, and 46% of the isolates, respectively, while SMX and KT had minimal FLC-sensitizing activity. Amikacin and DEX are efflux pump suppressors that permit the accumulation of FLC in yeast cells, and thus potentiate the FLC activity against FLC-R *Candida* spp. [21, 56]. On the other hand, COL triggers membrane permeabilization and cell death in ergosterol-depleted *Candida* cells due to azole treatment, while SMX inhibits the folate pathway and subsequently ergosterol biosynthesis in *Candida*, promoting synergy [20, 22]. Ketorolac was proven to be synergistic with FLC and was reported to repress fungal prostaglandins synthesis, biofilm development, and adhesion in *C. albicans* [23, 57].

To test the hypothesized inhibitory effects of AK and DEX on the activity of efflux pumps, the rhodamine efflux assay was performed using an FLC-R *C. albicans* (CA10) and an FLC-R NAC isolate (CT7). Amikacin suppressed the efflux pumps of CA10 and CT7, with percentages of reduction in the fluorescence intensity equivalent to 6.4% and 2% within two hours of exposure, respectively. Edward et al. reported that AK had a higher suppressing effect on the efflux pumps of *C. albicans* than NAC and suggested that the effect of AK is strain-dependent, which aligns with our results [21]. Dexamethasone possessed a higher inhibitory activity where it reduced the mean fluorescence intensity by 9.6% and 13% after 120 min when applied to CA10 and CT7, respectively. In accordance with our results, Sun et al. reported that DEX hindered rhodamine efflux in *C. albicans* [56].

The results of the checkerboard titration technique and resistance modulation assay pointed out that SMX and KT were the least promising repurposing agents; hence, they were excluded from the in vivo experiments, while the in vivo FLC-potentiating activities of AK, DEX, and COL were assessed using the bladder bioburden experiment. The administered doses of FLC, AK, DEX, and COL were chosen to be non-toxic in murine models [58–61]. The average count in the bladders indicated that the FLC/COL combination was the most effective, producing a 49.1% reduction in the bladder fungal burden relative to the control group. To the best of our knowledge, this is the first study to assess the in vivo efficacy of FLC combinations with AK, COL, or DEX against *Candida* in mice. The most relevant study we could retrieve reported that the caspofungin/COL combination produced a slight but significant reduction in the *Candida* burden in kidneys relative to caspofungin monotherapy [62]. We suggest that in vivo, DEX had an immunosuppressive effect, while AK induced an imbalance of the urinary microbial flora, promoting *Candida* predominance, which explains the failure of the FLC/DEX and FLC/AK combinations in vivo.

To further assess the efficacy of the successful combination (FLC/COL), we determined its impact on inflammation of the urinary bladder and the survival rate of *Candida*-challenged mice. Our results indicated that the FLC/COL reduced bladder inflammation by 42.9% relative to FLC or COL monotherapy, and by 50% relative to untreated mice. We believe that this is the first study demonstrating that combining COL with FLC caused a major reduction in the inflammatory responses of bladder tissues, to the extent that the bladders of combination-treated mice revealed near-normal histology. Moreover, the combination doubled the median survival rate compared to the FLC group and tripled it compared to the control and COL groups, which was corroborated by a single study reporting that FLC/COL increased the survival rate relative to FLC monotherapy in *Galleria mellonella* larva [22].

We acknowledge and understand the limitations of this study. These include the small number of tested *Candida* isolates and not testing the effect of the FLC/COL combination on the virulence factors of *Candida* spp. Moreover, the study of the virulence attributes at a molecular level is warranted. Further investigations into the dose-response and dose-adverse effect relationships of the combination are required to bring it a step closer to clinical application.

## Conclusions

The current study emphasizes the high prevalence of FLC resistance among urinary *Candida* spp., often complicated by a correlated elevation in virulence attributes. The results suggest that the FLC/COL combination offers a promising solution for UTIs caused by FLC-R *Candida* spp. as COL demonstrated an FLC-potentiating effect both in vitro and in vivo, where it not only reduced the bladder fungal burden and inflammation but also prolonged the survival of infected mice. Hence, repositioning the well-characterized COL as a sensitizer to FLC can be a compelling alternative to the high-cost, tedious, time-consuming process of novel antifungal drug discovery in the ongoing battle against antifungal resistance.

## Methods

### Collection and preservation of clinical isolates

A total of 34 *Candida* isolates were collected from the urine of patients admitted to Alexandria Main University Hospital, between October and December 2021. These isolates were stored in yeast extract-peptone-dextrose (YPD) broth containing 20% glycerol at -20 °C. Before use, a fresh culture was obtained by streaking an inoculum from the stock on Sabouraud Dextrose Agar (SDA, Millipore, Darmstadt, Germany), followed by incubation at 37 °C for 24 h to obtain separate pure colonies.

### Antimicrobial agents and chemicals

Fluconazole (Diflucan^®^, 2 mg/mL IV infusion), AK (Advomikacin^®^, 500 mg/2 mL IV/IM vial), DEX (Dexamethasone^®^, 8 mg/2 mL vial), and KT (Ketolac^®^, 30 mg/2 mL vial) were purchased from pharmaceutical markets. Colistin sulfate and SMX powders were obtained from Pharmacure Pharmaceutical Industries and Pharco Pharmaceuticals, Alexandria, Egypt, respectively. All stock solutions were prepared by dissolving the agent in sterile distilled water (DW). For the preparation of SMX stock solution, 1 N NaOH was added dropwise until the complete dissolution of the agent.

### Identification of *Candida* spp.

#### Germ tube formation test

Two to three colonies of each isolate were inoculated into tryptone soy broth (Himedia, Mumbai, India), incubated at 37 °C for 2 h, then examined using a magnification power of 100X on a microscopic slide with a cover slip for the detection of germ tubes. The elongated daughter cells emerging from the round mother cells without constriction at the origin were identified as germ tubes, while those with constriction at the origin were referred to as pseudo-hyphae. Germ tube positivity, characteristic for *C. albicans* and *C. dubliniensis*, was confirmed by the presence of at least five germ tubes in the entire mount. A negative result was indicated by the absence of germ tubes in a minimum of 10 fields [63]. Positive controls, *C. albicans* ATCC 231GI and ATCC 10231 were included in the experiment.

#### Tween 80 opacity test

The Tween 80 opacity test medium was prepared by the addition of 10 g bacteriological peptone (Lab M, Lancashire, UK), 5 g NaCl, 0.1 g CaCl_2_, and 15 g agar to 1 L of DW. After autoclaving, the medium was cooled to about 50 °C, and then 5 mL of autoclaved Tween 80 (Alpha Chemika, Mumbai, India) were incorporated. A few overnight colonies of each isolate were used to create a circular inoculation site of a 10 mm diameter on the agar plates. The plates were incubated at 30 °C and examined daily for 10 days under transmitted light for the presence of a halo zone around the inoculum, which was recorded as a positive result indicating the ability of the isolate to produce an esterase [64]. Inoculations were performed in duplicate.

#### Identification with Vitek^®^ 2 Advanced Expert System™

The identity of all isolates to the species level was confirmed using Vitek^®^ 2 Advanced Expert System™ (BioMérieux, Marcy l’Étoile, France) according to the manufacturer’s instructions [65].

### Phenotypic detection of the virulence attributes

#### Determination of the biofilm-forming ability by MTP method

A 100 µL-suspension of each tested isolate, prepared to match the turbidity of a 0.5 M McFarland standard (1 × 10^6^ to 5 × 10^6^ CFU/mL), was used to inoculate a well of a 96-well microtiter plate containing 100 µL of double-strength RPMI 1640 broth supplemented with L-glutamine and phenol red (Merck, Darmstadt, Germany), without NaHCO_3_, and buffered with morpholinopropane sulfonic acid (MOPS, Merck, Darmstadt, Germany). Following 48 h of incubation at 37 °C, planktonic cells were removed, and the wells were washed twice. Biofilms were then stained with 0.2% crystal violet, solubilized in 95% ethanol, and the absorbance was measured at 630 nm. The optical density of each strain (ODs) was compared to the absorbance of the negative control (ODnc), containing 100 µL sterile saline instead of yeast inoculum. The results were interpreted as follows: no biofilm formation (ODs ≤ ODnc), weak biofilm formation (ODnc < ODs ≤ 2 ODnc), moderate biofilm formation (2 ODnc < ODs ≤ 4 ODnc), and strong biofilm formation (4 ODnc < ODs) [21]. All isolates were tested in triplicate. Quality controls, *C. albicans* ATCC 231GI and ATCC 10231 were included in the experiment.

#### Production of proteinase enzymes

The ability of the tested isolates to produce proteinase enzymes was assessed using the method described by Edward et al. with few modifications [21]. Bovine serum albumin (BSA) medium was prepared using 2% dextrose, 0.1% KH_2_PO_4_, 0.05% MgSO_4_, 2% agar, and 1% BSA (Himedia, Mumbai, India), then 20 µL of yeast cells suspension (at a density of 10^6^ cells/mL) were dispensed into cups punched in the BSA medium. The plates were incubated at 37 °C for six days. The precipitation zone (Pz) value was calculated as the ratio of the diameter of the cup to the total diameter of the cup plus the precipitation zone, and the isolates were segregated accordingly into high producers (Pz = 0.35–0.5), moderate producers (Pz = 0.51–0.74), low producers (Pz = 0.75–0.9), and non-producers (Pz = 1). The test was performed in duplicate. *C. albicans* ATCC 231GI and ATCC 10231 were included in the experiment as quality controls.

#### Production of phospholipase enzymes

The egg yolk agar medium consisting of SDA, 1 M NaCl, 0.005 M CaCl_2_, and 8% sterile egg yolk emulsion (Himedia, Mumbai, India) was used to screen the isolates’ phospholipase activity. The egg yolk emulsion was centrifuged at 500 xg for 15 min at room temperature; the supernatant was completed to its initial volume with sterile DW and then added to the autoclaved medium. Twenty mL of the resultant medium were poured into each plate of 90 to 100 mm diameter. Aliquots of 20 µL of *Candida* suspension (approximately 10^6^ CFU/mL) were introduced into cups previously punched in the medium. The plates were incubated at 37 °C for 48 h, the Pz value was computed as the ratio between the diameter of the cup and the total diameter of the cup plus the precipitation zone, and the results were interpreted as follows: Pz < 0.63 indicates a strong enzymatic activity, 0.63 < Pz < 1.0, a moderate one, while Pz = 1 refers to no enzyme activity [21]. The test was performed in duplicate. Quality controls, *C. albicans* ATCC 231GI and ATCC 10231 were included in the experiment.

### Fluconazole susceptibility testing

The susceptibility of the isolates to FLC was determined using the DD method according to the performance standards for antimicrobial susceptibility testing of Clinical and Laboratory Standards Institute (CLSI) M44-Ed3 [66]. The FLC disks (25 µg, Himedia, Mumbai, India) were placed onto Müller-Hinton agar (Oxoid, Hampshire, UK) supplemented with 2% dextrose and 0.5 µg/mL methylene blue dye and inoculated with a suspension of yeast cells equivalent to 1 × 10^6^ to 5 × 10^6^ CFU/mL. The plates were incubated at 37 °C for 24–48 h. According to the diameters of the developed inhibition zones, *Candida* isolates were classified as S, SDD, or R following CLSI guidelines M27M44S-Ed3 [67]. Minimum inhibitory concentrations of FLC and repurposing agents: AK, COL, DEX, SMX, and KT were determined in the tested isolates using the BMD method in accordance with CLSI document M27-Ed4 [68]. A 50% inhibition in growth defined the MICs of FLC and KT [23, 67]. The MICs of DEX and SMX were indicated by 80% inhibition [20, 56], while 100% inhibition defined the MICs of AK and COL [21, 69]. Growth-positive and sterility control wells were included in each experiment, and *C. albicans* ATCC 231GI and ATCC 10231 were included as quality controls.

### Molecular characterization of FLC resistance mechanisms

#### Quantification of *ERG11* and efflux pump genes using RT-PCR

The Applied Biosystems 7500 Real-Time PCR System (Thermo Fisher Scientific Inc., Massachusetts, USA) was used to determine the expression levels of the *ERG11* gene as well as those of the efflux pump genes (*MDR1*,* CDR1*, and *CDR2*) in triplicate. Initially, RNA was extracted using TRIzol^®^ (Invitrogen™, Fischer Scientific, CA, USA) according to the manufacturer’s instructions, quantified by nanodrop One^c^ (Thermo Scientific, Massachusetts, USA), and then converted to cDNA using Topscript RT Drymix dN18/dN6 Kit (Enzynomics, Daejeon, Korea). Amplification of cDNA was carried out using primers obtained from Willowfort^®^, Birmingham, UK, for the efflux pump genes and from Eurofins genomics, Ebersberg, Germany, for the *ERG11* gene (Table 5). The composition of the reaction mixture and the cycling conditions are presented in Additional file 1: Table S5. To ensure the lack of primer-dimers, melting curves analysis was performed at 95 °C for 15 s followed by 50 °C for 1 min in the case of *MDR1*, *CDR1*, and *CDR2*, or 55 °C for 1 min in the case of *ERG11*. A representative example of melting curve analysis performed for the *CDR2* gene in *C. albicans* ATCC 10231 and 11 *Candida* spp. isolates is presented in Additional file 2: Fig. S3. The expression levels of all genes were normalized to the expression level of the housekeeping *ACT1* gene and compared to the expression levels in the FLC-S *C. albicans* ATCC 10231 using the 2^−ΔΔCt^ method.

**Table 5:** Primer pairs used for RT-PCR amplification and sequencing of the selected genes in this study

Target gene	Nucleotide sequence (5’ to 3’)	Amplicon size (bp)	Ref.
*ACT1*	F: TTGGTGATGAAGCCCAATCC R: CATATCGTCCCAGTTGGAAACA	86	[70]
*MDR1*	F: TTACCTGAAACTTTTGGCAAAACA R: ACTTGTGATTCTGTCGTTACCG	84	[70]
*CDR1*	F: TTTAGCCAGAACTTTCACTCATGATT R: TATTTATTTCTTCATGTTCATATGGATTGA	122	[70]
*CDR2*	F: GGTATTGGCTGGTCCTAATGTGA R: GCTTGAATCAAATAAGTGAATGGATTAC	81	[70]
*ERG11*	F: CCATTTGGTGGTGGTAGACA R: GGCACTTTATAACCATCAATAGTCC	119	Current study
*ERG11* (for seq.)	F: CACAGTTATAGACCCACAAGG R: TACTTAGCAACAACTTCTAGTG	1789	[71]
	IF: TATGAAAACTCAACCAGAAA	Binds at 564	Current study

*bp* base pair, *Ref.* reference, *seq.* sequencing, *F* forward primer, *R* reverse primer, *IF* internal forward primer

#### PCR amplification and sequencing of the *ERG11* gene

For the PCR amplification of the *ERG11* gene, DNA was extracted according to QIAamp^®^ DNA Mini and Blood Mini (Qiagen, Hilden, Germany) Handbook with a single modification, in which the zymolase enzyme was replaced with 0.2 g of 0.5 mm glass beads, followed by vortexing for 15 min [72]. The DNA concentration and purity were assessed before the amplification of the full-length *ERG11* gene (1587 bp), along with 71 bp upstream and 131 bp downstream of the gene using the forward and reverse primer pair indicated in Table 5. The composition of the reaction mixture and the cycling conditions are presented in Additional file 1: Table S5. The PCR product was electrophoresed on a 1% agarose gel to confirm that the amplicon was of the expected size through alignment with a 100 bp DNA ladder H3 RTU (Bio-helix Co. LTD, New Taipei City, Taiwan).

The PCR product was then purified according to the GeneJET Gel Extraction Kit’s (Thermo Scientific, MA, USA) manual and sequenced through primer elongation at three sites (forward, internal forward, and reverse primers, Table 5) using an ABI 3730xl sequencer (Thermo Fisher Scientific Inc., MA, USA) at GATC Biotech AG (Eurofins scientific, Cologne, Germany). The sequencing results were analyzed using BLAST^®^ (National Center for Biotechnology Information, NCBI, MD, USA) and FinchTV 1.4.0 (Geospiza Inc., WA, USA). The translated sequence was then compared with that of a previously published *ERG11* gene from an FLC-S strain available at NCBI (XM_002550939.1) using ProteinBLAST^®^. A representative example of the sequence analysis is shown in Additional file 2: Fig. S4.

### In vitro assessment of FLC interaction with potential repurposing agents

#### Checkerboard titration technique

The interaction of FLC with each of the five repurposing agents was assessed using the checkerboard titration technique against representative *Candida* isolates as previously described by Eldesouky et al. [73]. Fluconazole was tested in two-fold dilutions over a range of 500 to 0.5 µg/mL with AK (range, 32768 to 256 µg/mL), COL (range, 4096 to 32 µg/mL), DEX (range, 1000 to 8 µg/mL), SMX (range, 4096 to 32 µg/mL), and KT (range, 3750 to 32 µg/mL). The interactions between the tested drugs were determined by calculating the fractional inhibitory concentration index (ƩFICI), interpreted as follows: synergism (Syn); ƩFICI ≤ 0.5, additivity (Add); ƩFICI > 0.5 to ≤ 1, and indifference (Ind); ƩFICI > 1 and ≤ 4.

#### Resistance modulation assay

To evaluate the FLC resistance-modifying activity of the five repurposing agents, the MF was calculated as the ratio between the MIC of FLC alone and its MIC in the presence of a sub-inhibitory concentration of each agent, equivalent to 0.25X MIC against the most sensitive tested isolate [74]. An MF > 2 was set as the cut-off indicating significant resistance modulation [75].

#### Rhodamine efflux assay

To investigate whether AK and DEX influence the activity of efflux pumps in *Candida* isolates, the rhodamine efflux assay was conducted according to Edward et al. [21]. Cells were grown in YPD broth, harvested by centrifugation (6000 rpm at 4 °C for 5 min), washed with glucose-free phosphate-buffered saline (PBS), and their count was adjusted to 1 × 10^7^ cells/mL. Next, a 10 mM rhodamine solution (Loba Chemie, Mumbai, India) prepared in 95% ethanol was added to the *Candida* suspension to reach a final concentration of 10 µM [76]. The culture was incubated with rhodamine at 37 °C for 50 min and then kept on ice for 10 min. Cells were collected, washed with glucose-free PBS, and resuspended in 5% glucose/PBS. The tested drugs, AK and DEX, were added to reach a final sub-inhibitory concentration of 4096 and 250 µg/mL, respectively. A rhodamine-alone group (without drugs) served as a control. The fluorescence intensity of extracellular rhodamine was recorded by a spectrofluorometer (Shimadzu, Kyoto, Japan) at time intervals of 0, 30, 60, 90, and 120 min, with excitation at 485 nm and emission at 530 nm. For each result, an average value of three biological samples was used.

### In vivo assessment of combined therapy

A murine model was employed to evaluate the efficacy of the suggested combinations (FLC combined with AK, COL, or DEX) in vivo. Five-week-old female Swiss albino mice (18–22 g) were housed in animal rooms maintained at 23 ± 2 °C with 50 ± 20% relative humidity. The mice were divided into groups where food and water were provided *ad libitum*.

#### Organ bioburden experiment

The bladder bioburden was determined as previously reported by Mohamed et al. with some modifications [77]. Four mice were allocated into each of the following groups: control, FLC, repurposing agent (AK, COL, or DEX), and the combination group. The mice were challenged IP with a 200 µL injection of *C. albicans*, CA9 isolate, equivalent to 1 × 10^8^ to 3 × 10^8^ CFU/mouse. The inoculum of CA9 was prepared from an overnight culture in 0.9% NaCl containing 5% gastric mucin (Oxoid, Hampshire, UK). One hour post-infection, the control group was injected IP with 200 µL water for injection (WFI), the FLC group received 50 mg/kg FLC, the repurposing agent group received 40 mg/kg AK, 0.6 mg/kg DEX, or 5 mg/kg COL, while the combination group was treated with both FLC and the repurposing agent at the above-mentioned doses. Mice were sacrificed by cervical dislocation 24 h after treatment administration, and the bladder was dissected and homogenized. The fungal count in the bladder was then determined following serial dilution and cultivation on SDA plates in duplicate.

#### Animal survival experiment

To further assess the in vivo efficacy of the FLC/COL combination, animal survival was monitored according to Mohamed et al. with few modifications [78]. Briefly, four groups each containing six mice were infected IP with a 200 µL injection of CA9 isolate suspended in 0.9% NaCl solution containing 5% mucin at a dose equivalent to 1.5X the minimal lethal dose (MLD). This inoculum killed 100% of the untreated mice within a maximum of 48 h post-infection. One hour post-infection, the first group received 200 µL WFI, the second was treated with 50 mg/kg FLC, the third was injected with 5 mg/kg COL, and the fourth received 50 mg/kg FLC and 5 mg/kg COL simultaneously. All doses were administered IP and were repeated at 25 and 49 h post-infection. The survival of challenged mice was then observed for 7 days, and deaths were recorded.

#### Histopathological examination

To examine the tissue-protective activity of the FLC/COL combination, 16 mice challenged IP with a 200 µL injection of CA9 isolate equivalent to 1.5X MLD were segregated into four groups. One hour post-infection, the first group received 200 µL WFI, the second received 50 mg/kg FLC, the third was administered 5 mg/kg COL, and the fourth was treated with both FLC and COL IP at the mentioned doses. Mice were sacrificed 24 h post-infection, and their bladders were harvested and fixed in 10% buffered formalin. Three 5-mm-thick serial sections were cut from the paraffin-embedded tissue and stained with hematoxylin and eosin. The degree of inflammation in each section was examined by a histopathologist blinded to the sample origin and graded according to the criteria established by Hopkins et al. [79]. Bladders isolated from uninfected mice were assessed for comparative purposes.

### Statistical analysis

IBM^®^ SPSS^®^ statistics 25 program (IBM, NY, USA) was utilized to deduce the correlation between the resistance of the *Candida* isolates to FLC and their virulence, and the significance of such correlation through the calculation of r_s_ and the *p*-value. Microsoft Excel Spreadsheet Software (Microsoft, WA, USA) was used to perform the *t*-test to statistically analyze the RT-PCR results and detect significance in the gene expression levels. The *t*-test was applied as well to assess the significance of the reduction in the fluorescence intensity upon treatment with AK or DEX in the rhodamine efflux assay. For the analysis of the animal survival results, Kaplan Meier survival analysis and Log-rank (Mantel-Cox) test were performed using GraphPad Prism 9.5.1 (GraphPad Software, CA, USA). A *p*-value < 0.05 indicated statistical significance.

## Electronic supplementary material

Below is the link to the electronic supplementary material.

Additional file 1 shows Supplementary Tables S1 to S5.

Additional file 2 shows Supplementary Figures S1 to S4.

## Data Availability

The *ERG11* sequences generated during the current study can be freely and openly accessed at NCBI (https://www.ncbi.nlm.nih.gov/) under the accession numbers OR412823, OR412824, OR412825, and OR412826. The datasets supporting the conclusions of this article are included in this published article and its additional files.

## Abbreviations

BMD: Broth microdilution
CA: *C. albicans*
CD: *C. dubliniensis*
*CDR1*: *Candida* drug resistance-1
*CDR2*: *Candida* drug resistance-2
CF: *C. famata*
CG: *C. glabrata*
CT: *C. tropicalis*
DD: Disk diffusion
FLC: Fluconazole
FLC-R: FLC-resistant
FLC-S: FLC-sensitive
IP: Intraperitoneally
*MDR1*: Multidrug resistance-1
MLD: Minimal lethal dose
MTP: Microtiter plate
NAC: Non-albicans *Candida*
ODnc: Optical density of negative control
ODs: Optical density of strain
RPMI: Roswell Park Memorial Institute
r_s_: Spearman’s correlation coefficient
SDD: Susceptible-dose-dependent
WFI: Water for injection
