# Supplementary material for: Elucidation of the mechanisms of fluconazole resistance and repurposing treatment options against urinary Candida spp. isolated from hospitalized patients in Alexandria, Egypt
Source: BMC Microbiol. 2024 Oct 1;24:383. doi: 10.1186/s12866-024-03512-0 (PMC11443771; doi:10.1186/s12866-024-03512-0)
Supplement: Supplementary file 1 — Additional file 1 shows Supplementary Tables S1 to S5. [file 12866_2024_3512_MOESM1_ESM.docx]

**Supplementary information**

**Table S1:** Results of germ tube formation and tween 80 opacity tests for tested Candida spp. isolates.

| *Candida* spp. (n) | Number of isolates showing | | | | | |
| --- | --- | --- | --- | --- | --- | --- |
|  | Germ tube formation | Positive result in tween 80 opacity test after (days) | | | | Negative result in tween 80 opacity test |
|  |  | 2-3 | 4-5 | 6-8 | 9-10 |  |
| *C. albicans* (16) | 16 | 10 | - | 6 | - | - |
| *C. tropicalis* (10) | - | 7 | - | 3 | - | - |
| *C. glabrata* (6) | - | - | - | - | - | 6 |
| *C. famata* (1) | - | - | - | - | - | 1 |
| *C. dubliniensis* (1) | 1 | - | - | - | - | 1 |

**Table S2:** Inhibition zone diameters and MIC values of fluconazole against tested Candida spp. isolates.

| *Candida* spp. isolate | IZD (mm) | Interpretation | MIC (µg/mL) | Interpretation |
| --- | --- | --- | --- | --- |
| ATCC 231GI | 30 | S | 2 | S |
| ATCC 10231 | 39 | S | 2 | S |
| CA1 | 6 | R | 128 | R |
| CA2 | 11 | R | 8 | R |
| CA3 | 6 | R | >1000 | R |
| CA4 | 6 | R | 32 | R |
| CA5 | 6 | R | 256 | R |
| CA6 | 6 | R | 256 | R |
| CA7 | 6 | R | 32 | R |
| CA8 | 6 | R | 512 | R |
| CA9 | 6 | R | 1000 | R |
| CA10 | 6 | R | >1000 | R |
| CA11 | 22 | S | 2 | S |
| CA12 | 28 | S | 2 | S |
| CA13 | 32 | S | 4 | SDD |
| CA14 | 18 | S | 4 | SDD |
| CA15 | 17 | S | 1 | S |
| CA16 | 28 | S | 2 | S |
| CT1 | 6 | R | 128 | R |
| CT2 | 6 | R | 128 | R |
| CT3 | 6 | R | 128 | R |
| CT4 | 6 | R | 128 | R |
| CT5 | 6 | R | 128 | R |
| CT6 | 6 | R | 128 | R |
| CT7 | 6 | R | >1000 | R |
| CT8 | 6 | R | 256 | R |
| CT9 | 6 | R | 128 | R |
| CT10 | 6 | R | 128 | R |
| CG1 | 6 | R | 512 | R |
| CG2 | 15 | R | 64 | R |
| CG3 | 16 | SDD | 64 | R |
| CG4 | 11 | R | 64 | R |
| CG5 | 14 | R | 128 | R |
| CG6 | 17 | SDD | 32 | SDD |
| CF1 | 6 | R | 512 | R |
| CD1 | 30 | S | 1 | S |

*CA C. albicans, CT C. tropicalis, CG C. glabrata, CF C. famata, CD C. dubliniensis, IZD* inhibition zone diameter, *MIC* minimum inhibitory concentration, *S* susceptible, *SDD* susceptible-dose-dependent, *R* resistant

**Table S3:** MIC values of repurposing agents against fluconazole-resistant Candida spp. isolates.

| ***Candida* spp. isolate** | **MIC (µg/mL)** | | | | |
| --- | --- | --- | --- | --- | --- |
|  | AK | KT | COL | SMX | DEX |
| CA1 | >16384 | >2048 | 2048 | >2048 | >2000 |
| CA2 | >16384 | >2048 | 1024 | >2048 | >2000 |
| CA3 | >16384 | >2048 | 1024 | >2048 | >2000 |
| CA4 | >16384 | >2048 | 1024 | >2048 | >2000 |
| CA5 | >16384 | >2048 | 512 | >2048 | >2000 |
| CA6 | >16384 | >2048 | 128 | >2048 | >2000 |
| CA7 | >16384 | >2048 | >2048 | >2048 | >2000 |
| CA8 | >16384 | >2048 | >2048 | >2048 | >2000 |
| CA9 | >16384 | >2048 | 2048 | >2048 | >2000 |
| CA10 | >16384 | >2048 | 1024 | >2048 | >2000 |
| CT1 | 16384 | >2048 | 256 | >2048 | >2000 |
| CT2 | >16384 | >2048 | 256 | >2048 | >2000 |
| CT3 | >16384 | >2048 | 2048 | >2048 | >2000 |
| CT4 | >16384 | >2048 | 1024 | >2048 | >2000 |
| CT5 | >16384 | >2048 | 1024 | >2048 | >2000 |
| CT6 | >16384 | >2048 | 1024 | >2048 | >2000 |
| CT7 | >16384 | >2048 | 1024 | >2048 | >2000 |
| CT8 | >16384 | >2048 | 256 | >2048 | >2000 |
| CT9 | >16384 | >2048 | 256 | >2048 | >2000 |
| CT10 | >16384 | >2048 | 1024 | >2048 | >2000 |
| CG1 | >16384 | >2048 | >2048 | >2048 | >2000 |
| CG2 | >16384 | >2048 | 1024 | 2048 | 2000 |
| CG3 | >16384 | >2048 | >2048 | 2048 | >2000 |
| CG4 | >16384 | >2048 | 1024 | >2048 | >2000 |
| CG5 | >16384 | >2048 | >2048 | >2048 | >2000 |
| CF1 | >16384 | >2048 | >2048 | >2048 | >2000 |

CA C. albicans, CT C. tropicalis, CG C. glabrata, CF C. famata, MIC minimum inhibitory concentration, AK amikacin, KT ketorolac tromethamine, COL colistin sulfate, SMX sulfamethoxazole, DEX dexamethasone

**Table S4:** Modulation factors of repurposing agents for the fluconazole-resistant Candida spp. isolates.

| ***Candida* spp. isolate** | **Modulation factor** | | | | |
| --- | --- | --- | --- | --- | --- |
|  | AK | COL | DEX | KT | SMX |
| CA1 | 2 | 2 | 2 | 1 | 1 |
| CA2 | 1 | 1 | 1 | 1 | 1 |
| CA3 | 1 | 1024 | 256 | 128 | 128 |
| CA4 | 8 | 1 | 4 | 1 | 1 |
| CA5 | 1 | 1 | 1 | 1 | 1 |
| CA6 | 2 | 8 | 1 | 1 | 1 |
| CA7 | 4 | 32 | 64 | 1 | 1 |
| CA8 | 16 | 1 | 1024 | 4 | 16 |
| CA9 | 128 | 512 | 8 | 64 | 64 |
| CA10 | 256 | 1024 | 256 | 1 | 64 |
| CT1 | 2 | 2 | 1 | 1 | 1 |
| CT2 | 1 | 4 | 1 | 1 | 1 |
| CT3 | 1 | 4 | 1 | 1 | 1 |
| CT4 | 2 | 8 | 2 | 1 | 1 |
| CT5 | 1 | 1 | 1 | 1 | 1 |
| CT6 | 1 | 1 | 1 | 1 | 1 |
| CT7 | 16 | 128 | 8 | 1 | 1 |
| CT8 | 4 | 2 | 2 | 1 | 1 |
| CT9 | 2 | 2 | 1 | 1 | 1 |
| CT10 | 8 | 128 | 8 | 32 | 4 |
| CG1 | 2 | 2 | 256 | 2 | 2 |
| CG2 | 16 | 64 | 16 | 2 | 16 |
| CG3 | 4 | 1 | 64 | 1 | 2 |
| CG4 | 4 | 2 | 4 | 4 | 2 |
| CG5 | 16 | 2 | 256 | 1 | 1 |
| CF1 | 32 | 64 | 32 | 8 | 16 |

For the calculation of the modulation factor, minimum inhibitory concentrations ≥ 1000 µg/ml were approximated to 1024 µg/mL. CA C. albicans, CT C. tropicalis, CG C. glabrata, CF C. famata, AK amikacin, COL colistin sulfate, DEX dexamethasone, KT ketorolac tromethamine, SMX sulfamethoxazole

**Table S5:** Composition of reaction mixtures and cycling conditions used in RT-PCR and sequencing of the selected genes.

| **Target gene(s)** | **PCR reaction mixture** | **Cycling conditions** |
| --- | --- | --- |
| *CDR1*  *CDR2*  *MDR1* | - Topreal™ qPCR 2X premix (Enzynomics, Daejeon, Korea): 10 μL - F: 1 μL - R: 1 μL - Sterile nuclease-free water: 6 μL - cDNA: 2 μL | Initial denaturation: 95°C - 10 min  Denaturation: 95°C - 15 s  45 cycles  Annealing: 50°C - 15 s  Extension: 72°C - 30 s |
| *ERG11* |  | Initial denaturation: 95°C - 10 min  Denaturation: 95°C - 15 s  45 cycles  Annealing: 55°C - 15 s  Extension: 72°C - 30 s |
| *ERG11* (for sequencing)  *F* forward primer, *R* reverse primer, *WFI* water for injection | - 2X My Taq™ HS Red Mix (Bioline, London, UK): 25 μL - DNA extract: a volume equivalent to 400 – 600 ng DNA - F: 2 μL - R: 2 μL - WFI: added to 50 μL | Initial denaturation: 95°C - 10 min  Denaturation: 95°C - 1 min  40 cycles  Annealing: 51.7°C - 1 min  Extension: 72°C - 1.5 min  Final extension: 72°C - 10 min |
