# Supplementary material for: Elucidation of the mechanisms of fluconazole resistance and repurposing treatment options against urinary Candida spp. isolated from hospitalized patients in Alexandria, Egypt
Source: BMC Microbiol. 2024 Oct 1;24:383. doi: 10.1186/s12866-024-03512-0 (PMC11443771; doi:10.1186/s12866-024-03512-0)
Supplement: Supplementary file 2 — Additional file 2 shows Supplementary Figures S1 to S4. [file 12866_2024_3512_MOESM2_ESM.docx]

**Supplementary information**


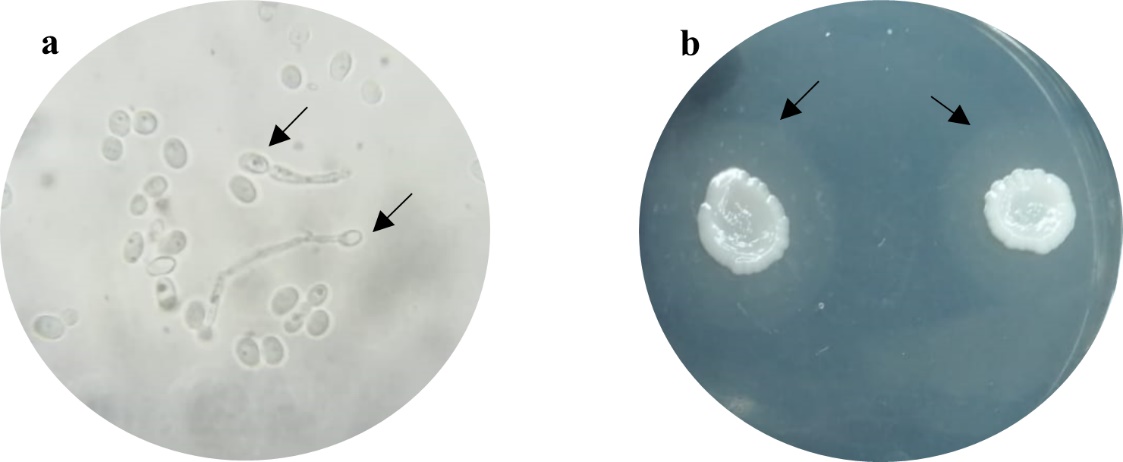


**Fig. S1:** **a** C. albicans ATCC 10231 showing germ tube formation; **b** Isolate CA10 showing halos around the inoculation sites in the tween 80 opacity test after 3 days.


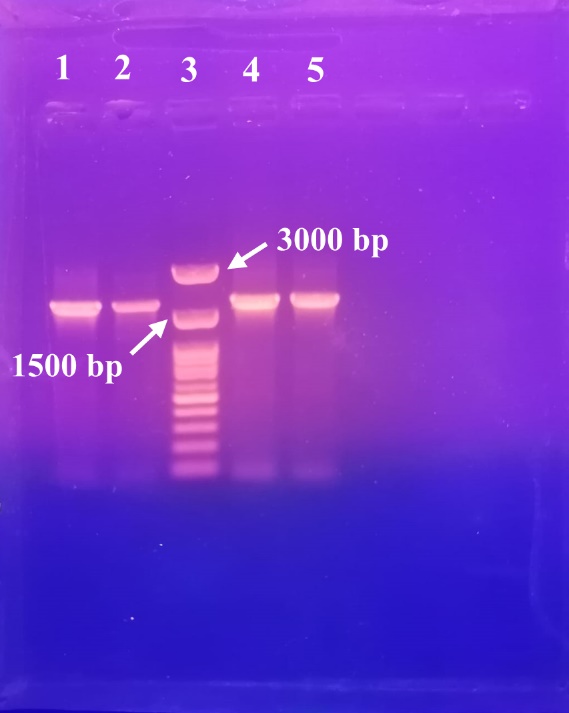


**Fig. S2:** Agarose gel showing PCR amplification of the *ERG11* gene in four *C. tropicalis* isolates. Lane 3 corresponds to a 100 bp DNA ladder. Lanes 1, 2, 4, and 5 show the amplicon (1789 bp) corresponding to *ERG11* gene in CT3, CT2, CT8, and CT1 isolates, respectively.


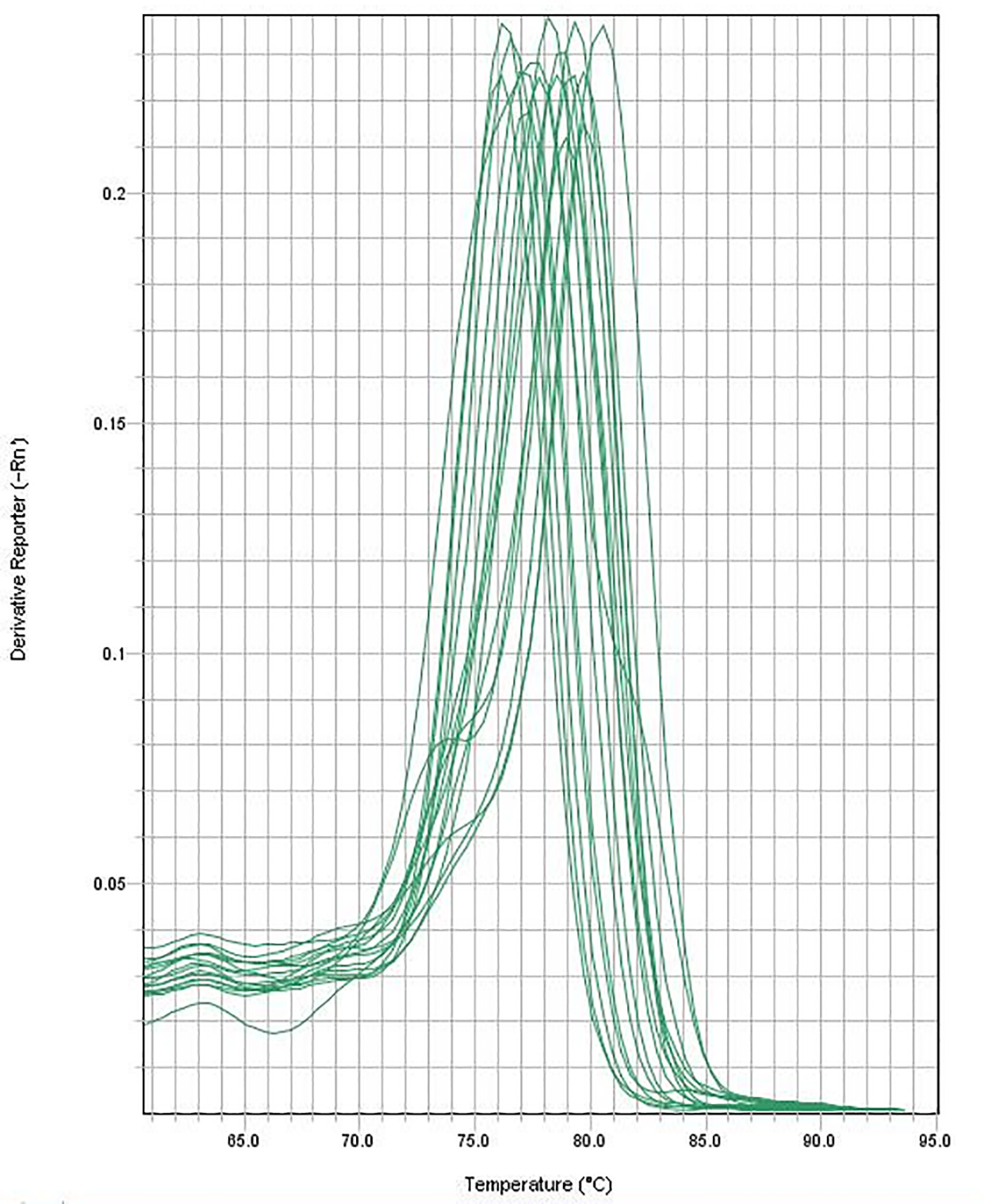


**Fig. S3:** Representative melting curve analysis for the efflux pump gene *CDR2* of *C. albicans* ATCC 10231 and 11 *Candida* spp. isolates.

**
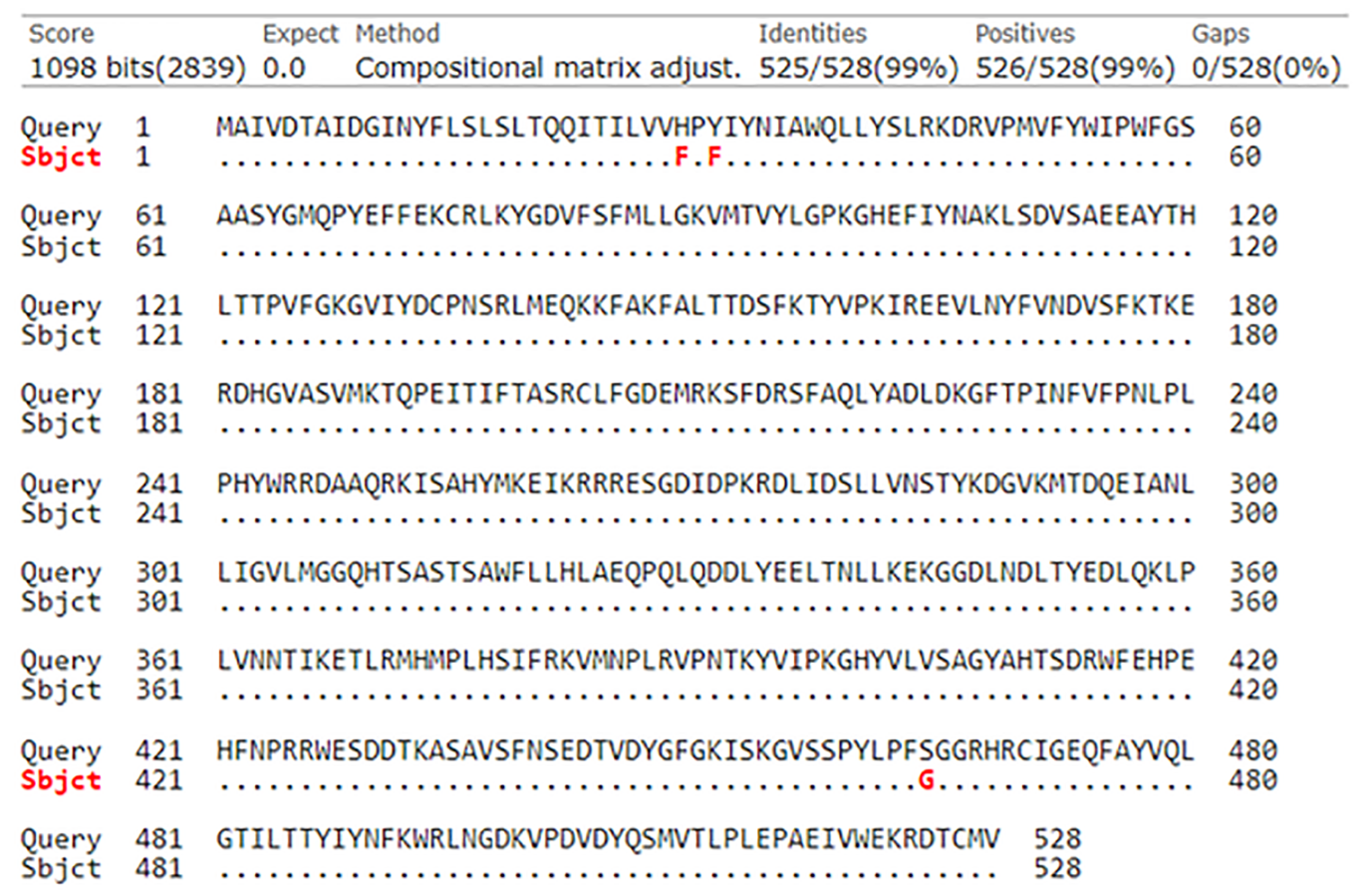
**

**Fig. S4:** *ERG11* sequence analysis in isolate CT1. Amino acid substitutions were detected through comparing the translated assembled *ERG11* gene of CT1 (*Query*) with that of an FLC-S strain ([XM_002550939.1](https://www.ncbi.nlm.nih.gov/nuccore/XM_002550939.1/) - *Sbjct*) using Protein BLAST . In this isolate, three amino acid substitutions (F28H, F30Y, and G464S) were detected.
